# Extramammary Paget's Disease: 20 Years of Experience in Chinese Population

**DOI:** 10.1155/2012/416418

**Published:** 2012-02-28

**Authors:** Jimmy Yu Wai Chan, George Kam Hop Li, Joseph Hon Ping Chung, Velda Ling Yu Chow

**Affiliations:** Division of Plastic & Reconstructive Surgery, Department of Surgery, University of Hong Kong Li Ka Shing Faculty of Medicine, Queen Mary Hospital, 102 Pokfulam Road, Hong Kong

## Abstract

*Background*. To examine the results of treatment of Extramammary Paget's disease (EMPD) in ethnic Chinese. *Method*. Between 1990 and 2010, patients treated for EMPD were reviewed. Data were analyzed retrospectively. *Results*. Forty-eight patients were treated by surgical resection. Local recurrence rate was 14.6%. The postresection defects were repaired by primary closure (8.3%), partial thickness skin graft (72.9%), or local/regional flaps (18.8%). Dermal invasion was found in 9 patients (18.8%). Seven patients (14.6%) developed regional lymph node metastasis (concurrent with surgery, *n* = 1; subsequent to surgery, *n* = 6), and 3 patients (6.3%) had systemic metastasis after surgery. The presence of dermal invasion was associated with significantly higher incidence of regional lymph nodes and systemic metastasis. The incidence of associated internal malignancy was 8.3%. *Conclusion*. The mainstay of treatment for EMPD is surgery. Pathological dermal invasion increases the chance of regional lymph node as well as systemic metastasis. The association with internal malignancy warrants preoperative endoscopic examination in all patients.

## 1. Introduction

Extramammary Paget's disease is an uncommon intra-epidermal carcinoma of apocrine gland-bearing skin. The most frequently involved anatomical sites include the scrotum, penis, vulva, and perineal and perianal region. Other rare sites of involvement, such as the eyelids [1], axilla, and external auditory canal [2], have been reported. There have been case reports describing ectopic EMPD occurring in areas devoid of apocrine gland, but they are exceedingly rare [3].

 In the majority of the cases, the disease is limited to the epidermis. However, it is well known that EMPD has the potential of dermal invasion [4]. Moreover, its association with underlying internal malignancies remained one of the most interesting characteristics of the disease. Data in the literature come from small series and case reports only, especially on Chinese patients.

## 2. Materials and Methods

 Between June 1990 and June 2010, all patients with EMPD, treated by the Division of Plastic and Reconstructive Surgery, Department of Surgery, Queen Mary Hospital, were reviewed. The diagnosis was confirmed by histological examination of incisional biopsy specimens. Ultrasound examination of the regional lymphatic drainage basins was performed for all patients before surgery, and any suspicious lymphadenopathy would be investigated by fine needle aspiration cytology. Special attention was paid to symptoms of possible associated gastrointestinal and urogenital malignancies. Routine endoscopic examination was performed preoperatively to rule out underlying malignancy.

All patients were treated by surgical resection under general anaesthesia. Lesions with well-defined borders were resected with 2 cm margins. For those tumours with indistinct borders, 3 cm resection margins were taken. Intraoperative frozen section technique was utilized in all patients to confirm margin clearance, and resection will continue until all resection margins were reported to be negative. The resultant wounds were either repaired by primary closure, split thickness skin graft, or local/regional flap reconstruction as indicated. Regional lymph node clearance was performed only when there was cytologically proven lymphatic metastasis. Postoperatively, all patients were followed up regularly at the outpatient clinic by experienced surgeons for possible loco-regional recurrence or systemic metastasis.

Data were collected from medical records and analyzed retrospectively. Statistical analyses were conducted using the Statistical Package for the Social Sciences (Windows version 18.0). A *P* value < 0.05 was regarded as statistically significant.

## 3. Results

 Between June 1990 and June 2010, 48 patients were referred to us for the management of EMPD. Among them, 41 were male and 7 were female. The age at presentation ranged from 51 to 89, with a mean age of 70 years old. The commonest presenting symptom was erythematous cutaneous lesion (*n* = 36), followed by ulceration (*n* = 9), groin lymph node enlargement (*n* = 1), and cutaneous mass (*n* = 2). Regions of involvement included penoscrotal area (*n* = 39), perianal/perineal region (*n* = 6), axilla (*n* = 2), and upper abdominal wall (*n* = 1). All patients presented initially to general practitioners, dermatologists, or urologists, with only 2 patients having their diagnosis correctly made and were referred to us immediately. The rest of the patients were misdiagnosed to have other dermatological conditions until subsequent biopsy of the lesion revealed the true diagnosis. The commonest misdiagnosis was eczema (82%), followed by contact dermatitis (12%), fungal infection (5%), and Bowen's disease (1%). The duration of delay before the correct diagnosis was made ranged from 6 to 48 months, with a median of 10 months.

Surgery was performed under general anaesthesia. All the cutaneous lesions were excised until frozen section examination of all the resection margins was negative. One patient had metastatic lymphadenopathy at the groin and simultaneous lymph node dissection was performed. Primary closure of the cutaneous defect was achieved only in 8.3% of the operations. Reconstruction by partial thickness skin grafts was required in 72.9% and local/regional flaps in 18.8% of the cases.

We observed 5 occasions (10.4%) when the initial frozen sections reports were negative but the subsequent final paraffin section results were positive. Further reexcisions were offered but only one of our patients agreed for reoperation. The remaining four patients developed local recurrence subsequently, while the operated patient remained disease-free after surgery.

The overall local recurrence rate was 14.6%. They were all treated by surgical salvage. The time to develop local recurrence ranges from 10 to 60 months, with median time being 24 months. There was a statistically significant relationship between local recurrence and positive resection margins at the initial surgery (*P* = 0.00014). However, it was not associated with decreased survival (*P* = 0.26).

Nine patients (18.8%) were found to have histological evidence of dermal invasion. However, invasive disease was not associated with increased chance of local recurrence (*P* = 0.44).

Seven patients (14.6%) developed regional lymph node metastasis, among which, six have the primary lesions showing histological evidence of dermal invasion. One patient had nodal metastasis on presentation, while the rest developed lymphatic metastasis after the initial surgery. All received lymph node dissection, except for one patient who was found to have systemic metastasis as well. Four patients with lymphatic metastasis ultimately died of the disease. There was a statistically significant relationship between lymph node metastasis and dermal invasion (*P* = 0.007) as well as mortality from the disease (*P* = 0.007).

Three patients (6.7%) developed systemic metastasis (liver, bone). One of them was found to have regional lymph node involvement as well as multiple liver metastases one year after initial surgery, and the remaining 2 patients developed bone metastasis 3 years after initial treatment. All of them died subsequently of the disease. The relationship between systemic metastasis and dermal invasion reached statistical significance (*P* = 0.002).

Six patients (nodal metastasis, *n* = 3; systemic metastasis, *n* = 2; nodal and systemic metastasis, *n* = 1) in our series ultimately died of the disease, and all of them had tumours showing dermal invasion. The mortality of patients with dermal invasion was 66.7%.  Table 1 summarized the relationship between dermal invasion and local recurrence after surgery, as well as the nodal/systemic metastasis and the mortality due to the disease.

Four patients (8.3%) were found to have associated internal malignancies at the time of work-up for the cutaneous lesion. Two patients presented with perianal EMPD, and carcinoma of the colon was found on colonoscopy. Wide excision of perianal lesion together with colectomy was done. The remaining patients have penoscrotal EMPD, and carcinoma of prostate was confirmed by transrectal biopsy. Excision of skin lesion and prostatectomy was done. However, one of them developed bone metastasis 3 years later and subsequently died of the disease. None of our patients was diagnosed to have internal malignancy after the initial investigation.

## 4. Discussion

**Figure 1:**
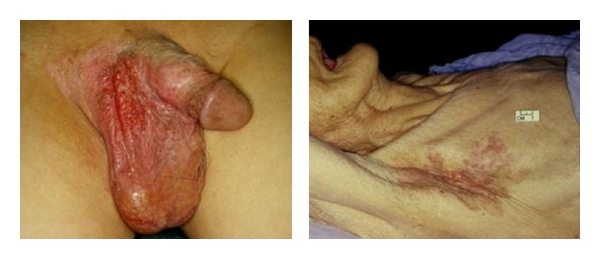
*(Left)* EMPD of the penoscrotal region. *(Right)* EMPD of the axilla. The disease usually presents as erythematous, “weepy” lesion, which often mimics benign inflammatory dermatological conditions, such as eczema.

 Extramammary Paget's disease is an uncommon intra-epithelial adenocarcinoma. It was originally described by Crocker in 1888 [5], fourteen years after Sir James Paget described the Mammary Paget's disease [6]. It occurs in areas of the body where apocrine glands are present (Figure 1). Clinically it often mimics benign inflammatory dermatological conditions, so it is not uncommon to have the diagnosis delayed until subsequent incisional biopsy confirms the true identity of the lesion. From the literature, the delay can be as long as 5−10 years [7]. The median time of delay before referral in our series is 10 months. This emphasizes the need for awareness of the condition among primary health care physicians, especially for those lesions occurring in the high-risk regions, which fail to respond to topical therapy. Biopsy of any suspicious lesions should be made so that proper referral to experienced surgeons can be made early.

**Figure 2:**
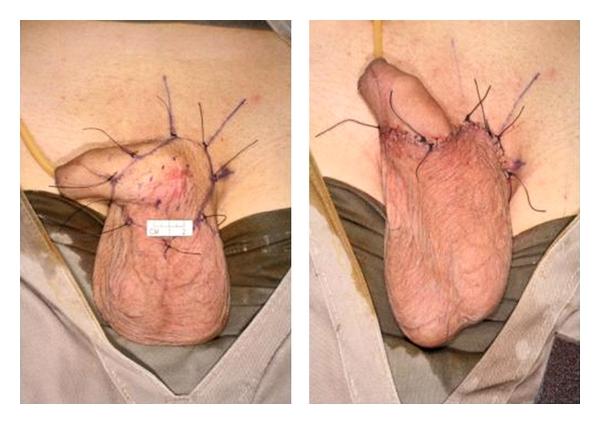
*(Left)* Small tumour involving penoscrotal region. The lesion was excised with 2 cm margins. *(Right)* Primary closure of resultant defect was possible utilizing the redundant scrotal skin.

**Figure 3:**
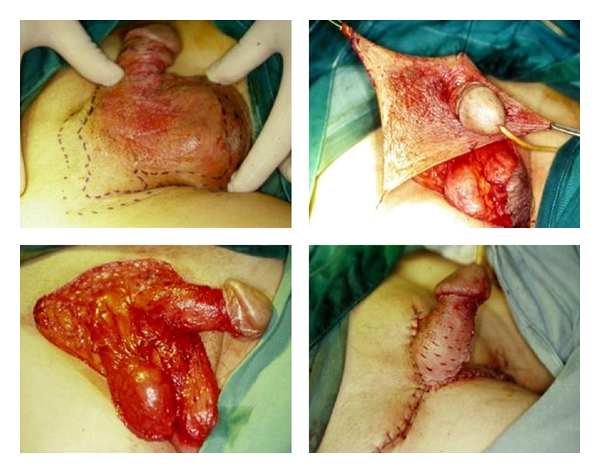
*(Above, left)* Large tumour involving penoscrotal region. *(Above, right)* Curative resection of the lesion required excision to the level of the corona of the glans penis. *(Below, left)* Resultant defect was too large for primary closure. *(Below, right)* Closure of the defect was achieved by mobilizing the upper thigh skin as well as partial thickness skin graft over the shaft of the penis.

**Figure 4:**
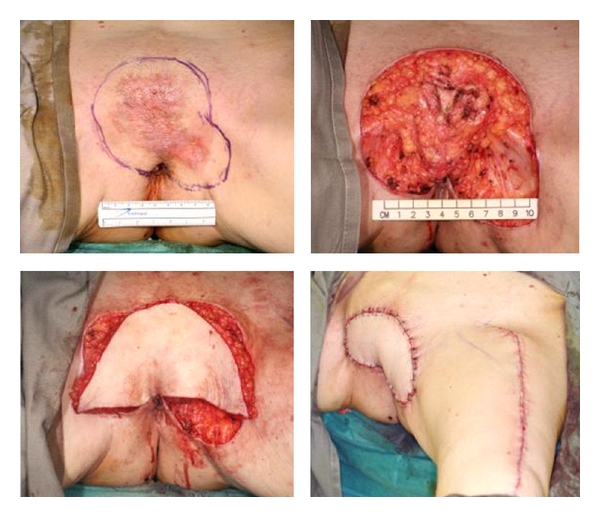
*(Above, left)* EMPD involving the pubic region. *(Above, right)* After resection, resultant defect measured 10 × 12 cm with underlying pubic ramus exposed. *(Below, left)* Pedicled anterolateral thigh perforator flap was harvested based on the perforators arising from the descending branch of lateral circumflex femoral artery. The flap then passed through a subcutaneous tunnel to reach the defect in the pubic region*. (Below, right)* Flap after inset and donor defect on the left thigh was closed primarily.

Complete surgical resection is the treatment of choice. Primary closure of the resultant defect is possible only in 8.3% of the occasions (Figure 2). Majority of the wounds required reconstruction using either skin graft (Figure 3) or local/regional flap (Figure 4). The extent of surgical resection is guided by intraoperative frozen technique. However, it is not always reliable. There has been a report showing that isolated tumour cells may extend well beyond the area of visible demarcation in an irregular pattern [8]. We have observed 5 occasions (10.4%) where there is discrepancy between frozen section and final paraffin section results. This may be explained by the fact that the correct diagnosis can often be reached only after careful evaluation of morphology with the help from a panel of immunohistochemical markers. We improve our technique of harvesting frozen section specimens by sending a continuous ring of tissue at the periphery and also a complete layer of tissue at the base of the surgical defect, thereby reducing the chance of sampling error. Others techniques, such as Mohs micrographic surgery [9–11], may help to improve the accuracy of mapping of the disease.

The local recurrence rate in our series was 14.6%, which is comparable with the reported range of 21% to 61% [12–14] in the literature. The median time to recurrence was 24 months, with one recurring 60 months after initial surgery. Long-term follow-up after surgery, therefore, is needed to detect possible recurrence.

It is known that a subgroup of patients may demonstrate dermal invasion. It is not clear whether these forms are the same disease in different stages of development or represent different disease entities. In our series, nine patients (18.8%) have evidence of dermal invasion on histological examination. Six of them developed regional lymph nodes metastasis, and four of them eventually died of the disease. The remaining patient with invasive disease was found to have bone metastasis and subsequently died. The prognosis of patients with invasive disease was poor, with a mortality of 66.7%. There was a statistically significant relationship between dermal invasion and regional lymph node metastasis (*P* = 0.007) and also systemic metastasis (*P* = 0.02). This is not difficult to understand, as the dermis harbors numerous lymphatic as well as blood vessels, which provide the routes for the tumour cells to metastasize. On the other hand, dermal invasion does not increase the risk of local recurrence (*P* = 0.44) in our series. This contradicts with the findings by Lai et al. [15], who believes that local recurrence is directly related to the pathological depth of invasion. Inadequate surgical resection with margins involvement, however, greatly increases the chance of local recurrence (*P* = 0.00014). Although local recurrence was not associated with decreased survival, further surgery is advisable for symptom control. Furthermore, it is still uncertain whether long standing local recurrent disease will transform to invasive form.

 EMPD may be associated with underlying anal and colorectal cancer [12] for perianal lesions and genitourinary malignancies [16, 17] for penoscrotal lesions. The reported incidence in Western literature ranged from 18% to 37% [12, 13, 16]. However, it appears that the incidence of associated malignancies in Asians is consistently lower than that in Caucasians [15, 18, 19]. The incidence in our series was 8.3%. Nevertheless, as these internal malignancies may remain asymptomatic at the time of diagnosis of the cutaneous lesion, routine endoscopic examination should be performed before surgery. Further and subsequent endoscopy may be necessary only in a selected group of patients with suspicious symptoms.

## 5. Conclusions

 Early diagnosis followed by curative resection and reconstruction remained the treatment of choice for EMPD. Dermal invasion is associated with poor prognosis. Although the association with underlying internal malignancy seems to be lower in Asian, routine preoperative endoscopic examination should be performed for every patient with follow-up endoscopy reserved for selected cases with suspicious symptoms.

## Figures and Tables

**Table 1:** 

	Carcinoma in situ	Dermal invasion	*P* value
	(*n* = 39)	(*n* = 9)	
Local recurrence	4	3	0.44
Nodal metastasis	1	6	0.007*
Systemic metastasis	0	3	0.002*
Mortality	0	6	0.001*
